# Supplementary material for: Iterative improvement in the automatic modular design of robot swarms
Source: PeerJ Comput Sci. 2020 Dec 7;6:e322. doi: 10.7717/peerj-cs.322 (PMC7924708; doi:10.7717/peerj-cs.322)
Supplement: Supplemental Information 3 [file peerj-cs-06-322-s003.zip › argos3/doc/api/standalone/a00546_source.html]

ARGoS: plugins/robots/mini-quadrotor/control\_interface/ci\_miniquadrotor\_rotor\_actuator.cpp Source File


- Main Page
- Related Pages
- Namespaces
- Classes
- Files

- File List
- File Members

# plugins/robots/mini-quadrotor/control\_interface/ci\_miniquadrotor\_rotor\_actuator.cpp

Go to the documentation of this file.

```
00001 
00007 #include "ci_miniquadrotor_rotor_actuator.h"
00008 
00009 #ifdef ARGOS_WITH_LUA
00010 #include <argos3/core/wrappers/lua/lua_utility.h>
00011 #endif
00012 
00013 namespace argos {
00014 
00015    /****************************************/
00016    /****************************************/
00017 
00018    CCI_MiniQuadrotorRotorActuator::SVelocities::SVelocities() {
00019       Velocities[0] = 0.0f;
00020       Velocities[1] = 0.0f;
00021       Velocities[2] = 0.0f;
00022       Velocities[3] = 0.0f;
00023    }
00024 
00025    /****************************************/
00026    /****************************************/
00027 
00028    CCI_MiniQuadrotorRotorActuator::SVelocities::SVelocities(Real f_north_vel,
00029                                                             Real f_west_vel,
00030                                                             Real f_south_vel,
00031                                                             Real f_east_vel) {
00032       Velocities[0] = f_north_vel;
00033       Velocities[1] = f_west_vel;
00034       Velocities[2] = f_south_vel;
00035       Velocities[3] = f_east_vel;
00036    }
00037 
00038    /****************************************/
00039    /****************************************/
00040 
00041    CCI_MiniQuadrotorRotorActuator::SVelocities::SVelocities(const SVelocities& s_velocities) {
00042       Velocities[0] = s_velocities.Velocities[0];
00043       Velocities[1] = s_velocities.Velocities[1];
00044       Velocities[2] = s_velocities.Velocities[2];
00045       Velocities[3] = s_velocities.Velocities[3];
00046    }
00047 
00048    /****************************************/
00049    /****************************************/
00050 
00051    CCI_MiniQuadrotorRotorActuator::SVelocities& CCI_MiniQuadrotorRotorActuator::SVelocities::operator=(const CCI_MiniQuadrotorRotorActuator::SVelocities& s_velocities) {
00052       if(&s_velocities != this) {
00053          Velocities[0] = s_velocities.Velocities[0];
00054          Velocities[1] = s_velocities.Velocities[1];
00055          Velocities[2] = s_velocities.Velocities[2];
00056          Velocities[3] = s_velocities.Velocities[3];
00057       }
00058       return *this;
00059    }
00060 
00061    /****************************************/
00062    /****************************************/
00063 
00064 #ifdef ARGOS_WITH_LUA
00065    /*
00066     * The stack must have four values in this order:
00067     * 1. north rotor velocity (a number)
00068     * 2. west rotor velocity (a number)
00069     * 3. south rotor velocity (a number)
00070     * 4. east rotor velocity (a number)
00071     */
00072    int LuaSetVelocity(lua_State* pt_lua_state) {
00073       /* Check parameters */
00074       if(lua_gettop(pt_lua_state) != 4) {
00075          return luaL_error(pt_lua_state, "robot.rotors.set_velocity() expects 4 arguments");
00076       }
00077       luaL_checktype(pt_lua_state, 1, LUA_TNUMBER);
00078       luaL_checktype(pt_lua_state, 2, LUA_TNUMBER);
00079       luaL_checktype(pt_lua_state, 3, LUA_TNUMBER);
00080       luaL_checktype(pt_lua_state, 4, LUA_TNUMBER);
00081       /* Perform action */
00082       CLuaUtility::GetDeviceInstance<CCI_MiniQuadrotorRotorActuator>(pt_lua_state, "rotors")->
00083          SetRotorVelocities(CCI_MiniQuadrotorRotorActuator::SVelocities(
00084                                lua_tonumber(pt_lua_state, 1),
00085                                lua_tonumber(pt_lua_state, 2),
00086                                lua_tonumber(pt_lua_state, 3),
00087                                lua_tonumber(pt_lua_state, 4)));
00088       return 0;
00089    }
00090 #endif
00091    
00092    /****************************************/
00093    /****************************************/
00094 
00095 #ifdef ARGOS_WITH_LUA
00096    void CCI_MiniQuadrotorRotorActuator::CreateLuaState(lua_State* pt_lua_state) {
00097       CLuaUtility::OpenRobotStateTable(pt_lua_state, "rotors");
00098       CLuaUtility::AddToTable(pt_lua_state, "_instance", this);
00099       CLuaUtility::AddToTable(pt_lua_state, "set_velocity", &LuaSetVelocity);
00100       CLuaUtility::CloseRobotStateTable(pt_lua_state);
00101    }
00102 #endif
00103 
00104    /****************************************/
00105    /****************************************/
00106 
00107 }
```

---

Generated on 10 Jul 2018 for ARGoS by 
 1.6.1
